# Allostatic Load Borne by Caregivers of Children with Serious Conditions: Acceptability of Self-Reported Data

**DOI:** 10.1177/26892820251390489

**Published:** 2025-10-24

**Authors:** Daniel H. Grossoehme, Sarah Friebert, Rachel Jenkins, Jonathan H. Pelletier, P. Cooper White, Michael L. Forbes

**Affiliations:** ^1^ Haslinger Family Pediatric Palliative Care Center, Akron Children’s Hospital, Akron, Ohio, USA.; ^2^ Rebecca D. Considine Research Institute, Akron Children’s Hospital, Akron, Ohio, USA.; ^3^ Department of Family & Community Medicine, Northeast Ohio Medical University, Rootstown, Ohio, USA.; ^4^ Department of Pediatrics, Northeast Ohio Medical University, Rootstown, Ohio, USA.; ^5^ Division of Critical Care Medicine, Akron Children’s Hospital, Akron, Ohio, USA.; ^6^ Akron Children’s Pediatrics, Akron, Ohio, USA.

**Keywords:** finance, health care utilization, palliative care, Pediatric

## Abstract

**Background:**

Stress due to a child’s serious medical condition affects the entire family. The cumulative stress burden (“allostatic load”) has deleterious health and economic effects.

**Objective:**

Demonstrate adequate caregiver willingness to disclose health care utilization and work/school attendance.

**Setting/Subjects:**

We enrolled caregivers of well children (WC), children with medical complexity (CMC), and children receiving palliative care (PC) (*N* = 15; 5 per group) in a midwestern United States pediatric hospital.

**Measurements:**

A novel allostatic load questionnaire and the Family Appraisal of Caregiving Questionnaire for Palliative Care were used.

**Results:**

All caregivers (100% female) completed the study, with 9% of data missing. PC participants reported greater child health care appointments and admissions compared with WC and CMC.

**Conclusions:**

Caregivers will disclose sensitive information for health care research, providing evidence for subsequent, adequately powered studies to inform policy-making. While underpowered for between-group analyses, results suggest pediatricians should routinely assess caregiver burden even at well-child visits.

## Key Message

Self-report and proxy disclosure of family health care utilization were acceptable, and results suggest foci for understanding family care burden, especially in families in which a child has a serious medical condition. These data are needed to guide policymaking and provide adequate support to families of children with significant conditions.

## Introduction 

Over one million U.S. children cope with life-limiting or life-shortening conditions. Resultant stress affects everyone, impacting their ability to function as family members, students, and employees.^1–3^ “Allostatic load” is defined as the cumulative burden of chronic stress and life events on bodies and minds; in short, the term refers to a condition’s impact across the range of an individual’s biopsychosocial functioning.^4–7^

While there are studies focused on specific health outcomes and direct hospital costs, limited attention has been given to the broader cumulative stress load families bear.^4,8^ Mitterer and colleagues described the financial and employment aspects of parents of children receiving palliative care (PC) in Switzerland.^9^ They quantified out-of-pocket medical expenses, averaging approximately USD $592 per month, though some spent nearly double that amount. Twenty-two percent of bereaved parents reported increased commitments at work, and 26% missed more than 30 days of work in the first year after their child’s death.^9^ Boyden and colleagues reported emotional and financial distress in a multisite sample of parents of children receiving PC in the United States.^10^ Financial distress is a construct reported and defined variously and without employing validated measures.^11^ In Boyden and colleagues’ multivariable model, family financial difficulty and child’s symptom score were individually associated with parental psychological distress. This is significant because children of parents with depressive or anxiety symptoms are more likely to have depressive or anxiety symptoms themselves.^10^ Nearly two-thirds (64%) of parents’ self-reported at least some financial difficulty, with 23% reporting “quite a bit” or “a great deal.” They also quantified the inability to pay rent or mortgage (22%), the possibility of food running out in the prior six months (22%), and being unable to purchase food in the prior six months (17%).^10^ Beyond financial stress, there is the time burden of providing care for a child with a serious medical condition, which can average nine hours per day of “hands-on” care.^12^ Adjustment to having a seriously ill sibling varies. However, siblings of the seriously ill child have more difficulty concentrating in school, anxiety, depression, guilt, lower physical well-being, and quality of life than those in families where all children are well.^13–17^ Clinical accounting of the extent to which a person’s or family’s stress load places them at risk for poor outcomes needs to be part of care plan development.^7^

Gaps remain in conceptualizing and measuring the sequelae of a child’s serious illness as the cumulative stress load borne by the family, economy, health care institution, and/or society. Until these knowledge gaps are filled, it will not be possible to authentically design, prototype, implement, and evaluate patient/family-centric models of care delivery to decrease the stress burden. The objective of this feasibility study was to demonstrate that participants would self-disclose information relating to their own (and their other children’s) work/school attendance, health care utilization, financial stressors, and other aspects of their allostatic load. This is a prerequisite for constructing a research agenda seeking to profoundly understand the breadth of caregivers’ allostatic load, with the ultimate goal of informing policy-making and guiding ambulatory care.^18^

## Methods

### Participants

This study was approved by the institutional review board of a 467-bed pediatric quaternary hospital system in the midwestern United States and carried out April–December 2024. A sample size of *N* = 15 was judged to be sufficient to demonstrate feasibility, operationalized as at least a 50% enrollment rate and less than 10% missing data. A large sample size was not required, as feasibility, not representativeness and generalizability, was the goal.^18–20^ Participants were recruited from one of three groups (*n* = 5 per group): caregivers of well children (WC), children with medical complexity (CMC), and children receiving PC. Having more than one comparison group increased the robustness of a posttest-only design.^20^ The use of WC as a comparison group minimized bias and confounding due to a child’s medical complexity.^20^ These groups were defined as follows. The American Academy of Pediatrics’ definition of CMC was used: chronic health conditions affecting multiple organ systems, functional limitations, high health care need/utilization, and use of or need for medical technology.^21^ This was operationalized using Feudtner and colleagues’ list of ICD-10 codes for CMC.^22^ “Well children” were defined as those seen by their primary care provider for a routine check-up. “Receiving PC” was defined as those followed by PC for long-term care (anticipated to be >12 months). “Sibling” was defined as any child, adolescent, or young adult, aged 6–21 years, residing with the index child, without regard to biological relationship, including other family members, foster children, or fictive kin. Inclusion criteria were: (1) caregiver (legal guardian) of a child with an ambulatory appointment in either the PC division or in a hospital system-owned pediatrician office providing primary care to both CMC and WC; (2) WC seen for a well-child primary care appointment and without any of the ICD-10 codes indicative of CMC; or (3) CMC having one of the ICD-10 codes indicative of CMC and ambulatory appointments in the prior 12 months in at least two other subspecialty clinics within the hospital system.^22^ Further, caregivers had to read and speak English sufficient to provide consent and participate and be the caregiver of at least two children, including the index child being seen in either clinic and at least one other between 6 and 21 years old and residing with the caregiver. Exclusion criteria were^1^ caregivers of an index child whose care began in the hospital system within the prior 12 months, or^2^ having had ambulatory care appointments in both PC and either WC or CMC clinics. STROBE guidelines were followed in the preparation of this article.^23^

### Procedures

Eligible patients were identified through chart review. Study staff notified the provider and/or the head nurse of the pediatric clinic of the patient’s potential eligibility. If the provider agreed the caregiver was eligible, the provider introduced the study to the caregiver. If the caregiver was interested, study staff discussed the study with the caregiver, answered questions, and, if indicated, proceeded with informed consent and data collection utilizing REDCap.^24^ Participants received a $10 gift card incentive after completing the study (typically 30 minutes). Clinical patient data were extracted from the patient’s electronic health record.

### Measure

No specific, validated measure of allostatic or cumulative stress load was available; thus, a study-specific questionnaire (Supplementary Table S1) was developed by the investigators to elicit health care utilization data for the index child, siblings, participant, and spouse partner (if any). Content validity was assessed by having the questionnaire completed by three caregivers followed by an informal cognitive interview. Also included was the Family Appraisal of Caregiving Questionnaire for Palliative Care (FACQ-PC).^25^ This 25-item measure has four subscales (caregiver strain, positive caregiving appraisals, caregiver distress, and family well-being; Cronbach’s alpha = 0.73–0.86) using a 5-point Likert-style scale (strongly disagree to strongly agree). Validated with adult caregivers of adults with cancer receiving PC, this tool has been used with caregivers of children and adolescents receiving pediatric PC and has demonstrated validity with comparison populations.^26,27^

### Analysis

Survey results were described with summary statistics, stratified based on respondent group (WC, CMC, or PC). As data were nonparametric with small cell counts, continuous variables were compared across groups using Kruskal–Wallis rank sum test, and categorical variables were compared using Fisher’s exact test. As the outcomes were feasibility and acceptability, there were no effect size estimates, and no power calculation was performed. When multiple siblings were present, questions regarding siblings were compared using the eldest sibling of the index patient, given the small number of families with multiple siblings. The FACQ-PC was scored according to its codebook.^25^ Cronbach’s alpha for the FACQ-PC was 0.7. All analyses were performed in R version 4.4.2 (R Foundation for Statistical Computing, Vienna, Austria). An alpha value of 0.05 defined statistical significance.

## Results

**FIG. 1.**
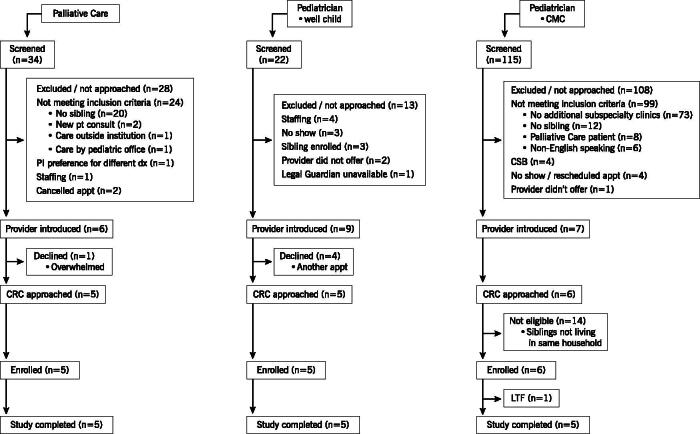
Study flow diagram.

Sixteen participants enrolled (94% enrollment rate; 100% female); demographic characteristics of their children are presented in Table 1 (see study flow diagram [Fig. 1]). Disclosure of participant health care utilization and caregiving appraisal was acceptable, evidenced by the missing data rate (9%) being less than the 10% threshold despite items not being required. Demographic and health care utilization data for participants’ children are presented in Table 1. Differences in the index child’s health care utilization suggest important differences among the three populations. Two items are of note concerning caregivers’ cumulative stress burden. First, participants with WC and PC children reported more difficulty concentrating at work or school compared with the CMC group (*p* = 0.031; see Supplementary Table S1). Second, of the three PC patients approved for home nursing, participants reported 48–68 unfilled hours/week in the prior three months. There were no other indications of meaningful differences in participants’ health care utilization, caregiving appraisal, nor between-group differences in the siblings of the index child (see Supplementary Tables S1, S2 and S3). Qualitative comments on ways participants, caregivers, and siblings were affected by the index child’s condition are presented in Table 2. Notably, comments come from PC and WC, but not CMC groups.

**Table 1.** Participant Gender and Index Child Health care Utilization Expressed as Median (Q1–Q3); Kruskal–Wallis Tests

Characteristic	Overall *N* = 15	Group			*p* value
		Well child *N* = 5	Child with medical complexity *N* = 5	Child with palliative care *N* = 5	
Child sex, *n* (%)					0.80^a^
Female	6 (40%)	1 (20%)	3 (60%)	2 (40%)	
Male	9 (60%)	4 (80%)	2 (40%)	3 (60%)	
Child race, *n* (%)					
African American/Black	10 (67)	3 (60)	2 (40)	5 (100)	
White	4 (27)	2 (40)	2 (40)	0 (0)	
Multiracial	1 (7)	0 (0)	0 (0)	0 (0)	
Child primary medical condition, *n* (%)					
Neurological	3 (20)	1 (20)	2 (40)	0 (0)	
Chromosomal/genetic	4 (27)	1 (20	3 (60)	0 (0)	
Prematurity	2 (13)	2 (40)	0 (0)	0 (0)	
Neurodegenerative	1 (7)	1 (20)	0 (0)	0 (0)	
None (well child)	5 (33)	0 (0)	0 (0)	5 (100)	
Child age (years), median (Q1, Q3)	11.1 (6.0, 15.0)	13.9 (10.4, 15.0)	11.1 (6.0, 11.5)	8.4 (1.6, 15.0)	0.40^b^
Number of appointments, median (Q1, Q3)	24 (10, 34)	4 (3, 10)	26 (24, 32)	43 (33, 48)	0.007^b,*^
Number of ED visits, median (Q1, Q3)	2 (0, 6)	2 (0, 6)	0 (0, 0)	5 (4, 8)	0.093^b^
Number of admissions, median (Q1, Q3)	0 (0, 1)	0 (0, 0)	0 (0, 0)	2 (1, 2)	0.019^b,*^
Length of stay (days), median (Q1, Q3)	0 (0, 3)	0 (0, 0)	0 (0, 0)	4 (3, 5)	0.016^b,*^
Number of ICU admissions, median (Q1, Q3)	0 (0, 0)	0 (0, 0)	0 (0, 0)	0 (0, 1)	0.12^b^
ICU length of stay (days), median (Q1, Q3)	0 (0, 0)	0 (0, 0)	0 (0, 0)	0 (0, 1)	0.12^b^

^a^ Fisher’s exact test.

^b^ Kruskal–Wallis rank sum test.

^*^ Significant at *p* ≤ 0.05.

**Table 2.** Qualitative Responses on Impact of Index Child’s Condition on Caregivers and Their Siblings (Unedited Verbatim Responses)

Question	Open responses (study group)^a^
Index child’s condition’s effect on participating caregiver:	Self-worth (PC)
	Pain/comfort level (PC)
	Emotionally (PC)
	Makes me very sad and depressed (WC)
	Behavioral (WC)
	Fear of unknown with the asthma severity (WC)
Index child’s condition’s effect on caregiver’s spouse/partner:	Emotionally (PC)
	Patience, understanding (WC)
Index child’s condition’s effect on their siblings:	Self-worth (PC)
	Feel neglected and not as wanted (PC)
	Not able to go to her games or pick up from practices (PC)
	They be sad his not feeling good (WC)
	Behavior (WC)
	Having to stay with other family members when sibling is hospitalized, getting to school, not getting attention to own needs

^a^ PC, Index child is palliative care; WC, Index child is a well child.

## Conclusion

This study presents results of a feasibility trial demonstrating questionnaire acceptability and identification of candidate outcome variables for subsequent studies. Despite the large number and potentially intimate nature of questionnaire items, participants in three settings voluntarily disclosed their data. Between-group differences suggest a greater family stress load for caregivers of children receiving PC due to that child’s health care utilization. The lack of unfilled nursing hours is an additional PC caregiver burden. Bearing in mind the small sample size, the difficulty concentrating at work reported by caregivers of WC—similar to that of PC caregivers—may be due to the WC clinic serving a population with a high proportion of families of minority race and/or low socioeconomic status, for whom the baseline cumulative stress load may frequently be high. Lower productivity due to on-the-job productivity losses (presenteeism), lost wages due to providing in-home care, and time away from home due to a child’s health care utilization carry a societal cost.^28^ WC participants’ narrative responses are notable for the allostatic load borne by this group, suggesting pediatricians should inquire of all families seen at routine visits about broader family health and coping. Unpacking cumulative stress allows providers to offer more tailored resources, strengthen resilience, and advocate for their patients and families in ways that extend outside office walls.

This feasibility study as limited by a small homogeneous sample (100% women, single center), which limits generalizability. There is the risk of provider gatekeeper (selection) bias, as eligible persons were only introduced to the study if their child’s provider agreed they were approachable. Nevertheless, important conclusions may be drawn about the feasibility of adequate engagement by caregivers to share this critical, sensitive information. The questionnaire content and length were acceptable, and family stress loads extend beyond health care bills. This study found a level of disclosure significant enough to merit further exploration for true implications on clinical and policy changes.

## Clinical Palliative Care Program

### Program structure

The PC division serves an independent 467-bed pediatric quaternary hospital system. Payment sources are philanthropy/grants, hospital support, and revenue from fee-for-service care provision with a small allocation from value-based arrangements.

### Team staffing

The team is comprised of the following disciplines: Physician (3.5 clinical FTE); Fellow Physician (2 FTE); Advanced Practice Provider (APP; 2 FTE); Nurse Case Manager (2 FTE); Social Worker (2.5 FTE); Bereavement Coordinator (1 FTE); Child Life Specialist (0.6 FTE); Dietitian (0.5 FTE); Chaplain (1.5 FTE); Rehabilitation Therapies (0.04 FTE); Scientist (0.5 FTE); Clinical Research Coordinator (0.6 FTE); Psychologist (0.25 FTE); Administrative & Operations Staff (4 FTE). The five dedicated PC physicians, one nurse case manager, and one APP have specialist PC certifications.

### Program availability

The division provides PC in a hospital, ambulatory clinic, and home (including facilities where patients reside); staff is available 24/7. Referral criteria individually established and agreed upon with several divisions (e.g., automatic Pediatric Intensive Critical Care Unit consult on admission >7 days; neuromuscular disorders beginning at age 12 if not needed before; Burn Center [end-of-life imminent or anticipated in 7 days, goals of care unclear, conflictual, or unarticulated; specialist bereavement care indicated, loss of functioning, on ventilator >7 days, SCORTEN ≥4; modified Baux score ≥100]; Maternal Fetal Medicine (MFM)/Fetal Treatment Center and Neonatal Intensive Care Unit for agreed-upon disorders; Oncology for all new diagnoses requiring chemotherapy or radiation; Bone Marrow Transplant patients on admission if not known prior; Hematology: sickle cell patients for parenteral pain management; and others).

### Patient volume and interactions

The PC division averages 3028 visits per year, with an average daily census of 35 inpatients (seen on average three times per week) and 1000 active patients overall, who have an average length of stay in PC of 1432 days.

## Abbreviations Used

APP: Advanced Practice Provider
CMC: children with medical complexity
FTE: full-time equivalent
MFM: Maternal Fetal Medicine
PC: Palliative Care
USD: United States Dollars
U.S.: United States
WC: Well child
