# Supplementary material for: Allostatic Load Borne by Caregivers of Children with Serious Conditions: Acceptability of Self-Reported Data
Source: Palliat Med Rep. 2025 Oct 24;6(1):521–6. doi: 10.1177/26892820251390489 (PMC12670708; doi:10.1177/26892820251390489)
Supplement: Supplementary Tables [file 26892820251390489_suppl_tables.docx]

**Supplemental Table 1. Participants’ missed work and school**

| **Work/School items** | **Overall** N = 15 | **Group** | | | **p-value** |
| --- | --- | --- | --- | --- | --- |
|  |  | **LP Well Child** N = 5 | **LP CMC** N = 5 | **Palliative Care** N = 5 |  |
| Days of Missed Work or School in Last 3 Months, Median (Q1, Q3) [Min, Max] | 5 (3, 6) [0, 15] | 6 (3, 7) [0, 7] | 3 (2, 4) [2, 4] | 11 (6, 15) [6, 15] | 0.16*^1^* |
| Unknown | 6 | 1 | 2 | 3 |  |
| Because of your child's condition, when you are at work or school, how easy or hard is it to concentrate on your job? Median (Q1, Q3) [Min, Max] | 5 (3, 6) [0, 9] | 4 (3, 6) [2, 6] | 7 (6, 8) [5, 9] | 4 (2, 5) [0, 5] | 0.031*^1^* |
| Unknown | 2 | 1 | 0 | 1 |  |
| Because of your child's condition, have you changed jobs or reduced the number of hours at your job? n (%) |  |  |  |  | 0.27*^2^* |
| Yes | 6 (40) | 2 (40) | 2 (40) | 2 (40) |  |
| No | 5 (33) | 2 (40) | 3 (60) | 0 (0) |  |
| Not Applicable | 4 (27) | 1 (20) | 0 (0) | 3 (60) |  |
| Other than a routine checkup ('physical') by your primary doctor, how many times have you been to see a doctor or Nurse Practitioner for a problem or question? Median (Q1, Q3) [Min, Max] | 2 (0, 3) [0, 10] | 0 (0, 3) [0, 6] | 2 (1, 3) [0, 5] | 2 (1, 3) [0, 10] | 0.75*^1^* |
| How many times have you been to an urgent care clinic for your own health needs? Median (Q1, Q3) [Min, Max] | 1 (0, 2) [0, 6] | 0 (0, 1) [0, 6] | 0 (0, 1) [0, 1] | 2 (1, 2) [0, 3] | 0.24*^1^* |
| How many times have you been to an Emergency Department for your own needs? Median (Q1, Q3) [Min, Max] | 0 (0, 1) [0, 5] | 1 (0, 1) [0, 5] | 0 (0, 0) [0, 1] | 0 (0, 0) [0, 1] | 0.27*^1^* |
| Other than your primary care doctor, how many times have you been to an outpatient or clinic appointment for your own needs? Median (Q1, Q3) [Min, Max] | 0 (0, 4) [0, 10] | 0 (0, 5) [0, 10] | 0 (0, 0) [0, 5] | 1 (0, 2) [0, 4] | 0.67*^1^* |
| How many times have you been to see a mental health provider for your own needs? Median (Q1, Q3) [Min, Max] | 0 (0, 6) [0, 6] | 0 (0, 0) [0, 6] | 1 (0, 6) [0, 6] | 0 (0, 6) [0, 6] | 0.56*^1^* |
| How many times have you been admitted to a hospital overnight? Median (Q1, Q3) [Min, Max] | 0 (0, 0) [0, 3] | 1 (0, 1) [0, 3] | 0 (0, 0) [0, 0] | 0 (0, 0) [0, 0] | 0.031*^1^* |
| How many times have you been in a partial hospitalization or intensive outpatient psychiatric program? Median (Q1, Q3) [Min, Max] | 0 (0, 0) [0, 0] | 0 (0, 0) [0, 0] | 0 (0, 0) [0, 0] | 0 (0, 0) [0, 0] |  |
| *^1^* Kruskal-Wallis rank sum test | | | | | |
| *^2^* Fisher’s exact test | | | | | |

**Supplemental Table 2. Missed school by index child and siblings**

| **Question** | **Overall** N = 8 | **LP Well Child** N = 4 | **LP CMC** N = 2 | **Palliative Care** N = 2 | **p-value***^1^* |
| --- | --- | --- | --- | --- | --- |
| Over the past 3 months how many days of school has your child missed because of illness? Median (IQR) | 7 (3 – 15) | 8 (4 – 15) | 6 (2 – 9) | 12 (2 – 21) | 0.70 |
| Over the past 3 months how many days of school has your child's sibling missed because of illness? Median (IQR) | 0 (0 – 1) | 0 (0 – 1) | 0 (0 – 0) | 1 (0 – 1) | 0.63 |
| *^1^* Kruskal-Wallis rank sum test | | | | | |

**Supplemental Table 3. Home and school items for index child and sibling**

| **Question** | **Overall** N = 15 | **LP Well Child** N = 5 | **LP CMC** N = 5 | **Palliative Care** N = 5 | **p-value** |
| --- | --- | --- | --- | --- | --- |
| Does your child receive home nursing? n (%) | 3 (20) | 0 (0) | 0 (0) | 3 (60) | 0.066^1^ |
| Over the past three months, on average, how many hours per week have you not had nursing in vour home for your child? Median (Q1, Q3) [Min, Max] | 96 (48, 168) [48, 168] | N/A | N/A | 96 (48, 168) [48, 168] |  |
| Unknown | 12 | 5 | 5 | 2 |  |
| To what extent does the time you spent caring for the child who was seen in clinic today limit the time you were able to spend with other children? Median (Q1, Q3) [Min, Max] | 5 (2,7) [0,10] | 5 (0.7) [0.7] | 6 (5.7) [2,7] | 5 (4,6) [0,10] | 0.78^2^ |
| To what extent does having to limit your time with your other children lead you to feel guilty? Median (Q1, Q3) [Min, Max] | 5 (4,7) [1,10] | 7 (5,7) [5.7] | 5 (3,6) [0.7] | 5 (4,8) [4,10] | 0.45^2^ |
| Unknown | 3 | 2 | 0 | 1 |  |
| Has your child received detention at school? n (%) | 4 (50) | 4 (80) | 0 (0) | 0 (0) | 0.14*^1^* |
| Unknown | 7 | 0 | 3 | 4 |  |
| Has your child been suspended from school? n (%) | 1 (13) | 1 (20) | 0 (0) | 0 (0) | >0.99*^1^* |
| Unknown | 7 | 0 | 3 | 4 |  |
| Has your child been to an urgent care clinic? n (%) | 3 (20) | 1 (20) | 0 (0) | 2 (40) | 0.73*^1^* |
| Has your child been to the Emergency Department? n (%) | 6 (40) | 3 (60) | 0 (0) | 3 (60) | 0.13*^1^* |
| Has your child been admitted to the hospital? n (%) | 4 (27) | 1 (20) | 0 (0) | 3 (60) | 0.23*^1^* |
| Has your child been in a partial hospitalization or intensive outpatient psychiatric program? n (%) | 3 (20) | 1 (20) | 1 (20) | 1 (20) | >0.99*^1^* |
| Has your child's sibling been to an urgent care clinic? n (%) | 4 (27) | 2 (40) | 0 (0) | 2 (40) | 0.45*^1^* |
| Has your child's sibling been to the Emergency Department? n (%) | 5 (33) | 3 (60) | 0 (0) | 2 (40) | 0.25*^1^* |
| Has your child's sibling needed psychiatric care? n (%) | 8 (53) | 1 (20) | 3 (60) | 4 (80) | 0.30*^1^* |
| *^1^* Fisher’s exact test  *^2^* Kruskal-Wallis rank sum test | | | | | |
